# Moisture parameters and fungal communities associated with gypsum drywall in buildings

**DOI:** 10.1186/s40168-015-0137-y

**Published:** 2015-12-08

**Authors:** Sandra Dedesko, Jeffrey A. Siegel

**Affiliations:** Department of Civil Engineering, University of Toronto, 35 St George St, Toronto, ON M5S 1A4 Canada; Dalla Lana School of Public Health, University of Toronto, 223 College Street, Toronto, ON M5T 1R4 Canada

**Keywords:** Relative humidity, Fungi, Moisture content, Equilibrium relative humidity, Time of wetness, Microorganisms

## Abstract

Uncontrolled excess moisture in buildings is a common problem that can lead to changes in fungal communities. In buildings, moisture parameters can be classified by location and include assessments of moisture in the air, at a surface, or within a material. These parameters are not equivalent in dynamic indoor environments, which makes moisture-induced fungal growth in buildings a complex occurrence. In order to determine the circumstances that lead to such growth, it is essential to have a thorough understanding of in situ moisture measurement, the influence of building factors on moisture parameters, and the levels of these moisture parameters that lead to indoor fungal growth. Currently, there are disagreements in the literature on this topic. A literature review was conducted specifically on moisture-induced fungal growth on gypsum drywall. This review revealed that there is no consistent measurement approach used to characterize moisture in laboratory and field studies, with relative humidity measurements being most common. Additionally, many studies identify a critical moisture value, below which fungal growth will not occur. The values defined by relative humidity encompassed the largest range, while those defined by moisture content exhibited the highest variation. Critical values defined by equilibrium relative humidity were most consistent, and this is likely due to equilibrium relative humidity being the most relevant moisture parameter to microbial growth, since it is a reasonable measure of moisture available at surfaces, where fungi often proliferate. Several sources concur that surface moisture, particularly liquid water, is the prominent factor influencing microbial changes and that moisture in the air and within a material are of lesser importance. However, even if surface moisture is assessed, a single critical moisture level to prevent fungal growth cannot be defined, due to a number of factors, including variations in fungal genera and/or species, temperature, and nutrient availability. Despite these complexities, meaningful measurements can still be made to inform fungal growth by making localised, long-term, and continuous measurements of surface moisture. Such an approach will capture variations in a material’s surface moisture, which could provide insight on a number of conditions that could lead to fungal proliferation.

**Electronic supplementary material:**

The online version of this article (doi:10.1186/s40168-015-0137-y) contains supplementary material, which is available to authorized users.

## Background

Uncontrolled moisture in buildings can lead to a number of problems. Indoor moisture can originate from many sources, including transportation from the outdoors by vapour diffusion through the building envelope, groundwater intrusion, and penetration of precipitation [1]; indoor activities, such as cooking, showering, and cleaning [2]; and building design and/or operational issues, such as plumbing leaks and uncontrolled airflows [3]. Such occurrences can result in a number of problems, including structural damage, material degradation, health concerns, and changes to microbial communities [3–7]. A study conducted by the Canada Mortgage and Housing Corporation (CMHC) [8] found that an increase in moisture content (MC) from 0 to 2 % caused a dramatic decrease in the flexural strength and resistance to fastener pull-through of gypsum drywall, and a MC of around 5 % caused the specimens to crumble. Adan and Samson [9] noted that changes in a material’s moisture can result in efflorescence and discoloration caused by the evaporation of liquids and crystallization of dissolved salts, spalling, and cracking. More concerning, however, is the positive association between indoor dampness and allergic and respiratory symptoms and diseases [10]. Although the comprehensive effects of damp buildings on occupant health are still unclear [11], there have been accounts of both minor and severe moisture-induced health symptoms, with a rare case of the latter being an epidemiologic association between pulmonary haemorrhaging in small children and water-damaged homes containing toxic fungi [12, 13]. The predominant fungi in this investigation was *Stachybotrys*, which requires wetted cellulose, a common component of building materials, to grow [14] and has been found growing on gypsum drywall in other buildings with moisture damage [15]. Furthermore, fungal growth and the number of different actively growing fungal species have been shown to increase with moisture [16], and Allsopp and colleagues [17] noted that building materials are susceptible to various mechanisms of biodeterioration (e.g. rot) from such microorganisms.

Evidently, moisture and microbial communities have a pronounced impact on the built environment, and accordingly, this has been the focus of many studies. Due to the complexity of this topic, there is inconsistency in the literature related to (1) moisture assessment in buildings, (2) acceptable levels of indoor moisture, and (3) how indoor moisture affects microbial growth. Beginning with the first inconsistency, the approaches used to characterize moisture (e.g. measurement device, parameter, frequency, and location) differ in laboratory and field studies of moisture-induced microbial growth. This can be problematic when interpreting moisture levels and comparing results from different studies because different measurement devices [8] and techniques [18] can result in different levels and interpretations of moisture. Second, there is a lack of agreement on acceptable levels of indoor moisture in the literature. The U.S. Environmental Protection Agency [19] recommends indoor relative humidity (RH) be maintained between 30 and 50 % (with a maximum limit of 60 %), while the International Energy Agency [20] states a maximum indoor RH of 80 %. ASHRAE specifies indoor moisture levels for ventilation systems with dehumidification capabilities, including a maximum indoor RH of 65 % in Standard 62.1-2013 [21] and a dewpoint temperature of 16.8 °C to ensure occupant comfort in Standard 55-2013 [22]. In actuality, such levels are difficult to consistently achieve, since moisture in buildings is dynamic and difficult to control, which can result in unwanted problems, such as microbial growth. This leads to the third inconsistency: the lack of agreement on a critical moisture value that if not reached or exceeded, should prevent microbial growth. The values defined in the literature encompass a large range, which is likely due to the differences in the methodologies used to facilitate microbial growth, assess moisture, and characterize microbial communities. Many studies analysed microbial communities on building materials under controlled moisture conditions in the laboratory, but used methodologies, such as artificial inoculations [23] and exposure to constant hygrothermal conditions [24, 25], that are unrepresentative of most operating buildings. Furthermore, some studies investigated microbial communities in moisture-prone areas of residences [26] and water-damaged buildings [27–29], but did not include any quantitative assessment of moisture. The studies that did quantitatively measure moisture often assessed different moisture parameters, which are not always comparable or equivalent. Lastly, the methods used to analyse microbial communities differed, as some studies utilized molecular techniques [26] while others relied on a culture-based approach [24]. This can be problematic when interpreting results, as these methods have been shown to produce different community characterizations (e.g. [30]). Several researchers experienced difficulties detecting certain fungal taxa, such as *Aspergillus* and *Penicillium*, with molecular methods (e.g. [26, 31]), while others have noted that not all species will grow on a specific culture medium [30]. Pietarinen and colleagues [30] found that molecular- and culture-based approaches yielded different concentrations of certain fungi, and that certain species were detected exclusively by either the culture or molecular method, depending on concentration. Andersen and colleagues [32] addressed these difficulties and stated that all methods are biased in some way and that currently, there is no single method that can provide a complete characterization of the microbial community under investigation. Although numerous studies have addressed moisture-induced microbial growth, the variation in methodologies and critical moisture values prevents a comprehensive understanding of the moisture level that will lead to microbial growth in buildings.

### Review scope and objectives

Overall, the literature pertaining to moisture-induced microbial proliferation in buildings is yet to reach consensus on in situ moisture measurement, moisture levels in buildings, and moisture-induced microbial growth indoors. Accordingly, this brings a number of research questions for this review, which include:

1. How is moisture measured in buildings?
2. How do building factors, such as material properties, building assemblies, and indoor environmental conditions, influence moisture?
3. What impact does indoor moisture have on indoor fungal communities and fungal growth?

This review paper discusses the above questions with a specific focus on moisture-induced fungal growth on gypsum drywall in buildings. Although excess moisture has been shown to result in both fungal and bacterial growth, the scope of this review paper is generally limited to moisture-induced fungal growth because this is what the majority of the literature describes. Furthermore, gypsum drywall (which is sometimes referred to as wallboard, plasterboard, or gypsum board) was selected as the material of interest because:

- It is a ubiquitous building material used in both existing and new constructions [33].
- It is not intended to get wet, but it is very sensitive to moisture [34].
- It has a pronounced behaviour in response to moisture that is distinct from other building materials. Its surface moisture has been found to be most similar to ambient conditions during periods of increasing humidity and least similar to ambient conditions during decreasing humidity compared to other common interior finishing materials (i.e. ceiling tile and carpet) [35, 36].
- There have been several cases where it was the specific site of fungal growth in buildings due to its properties and location in building assemblies [37, 38].

Narrowing the scope of this review to fungal proliferation on a single material allows for a deeper analysis within a broad and complex general topic. This review begins with a more general discussion of moisture measurement in buildings, followed by drywall-specific discussions of how moisture is affected by building factors, and what implications this has on fungal communities.

## Review

### Research question 1: how is moisture measured in buildings?

**Fig. 1:**
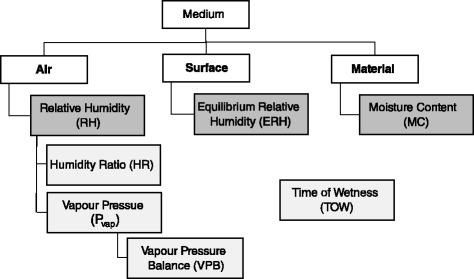
Classification of *in-situ* moisture parameters by measurement location in buildings. Measureable parameters are shown in *dark grey boxes* below the various locations, and inferred parameters are shown in *light grey boxes* near the measureable parameter they are inferred from. TOW is not positioned under a single measurement location because it can be calculated for all three locations and all parameters; although, it is traditionally applied to *a*_w_

**Fig. 2:**
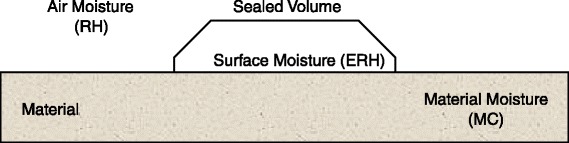
Visual schematic of the three locations in buildings where moisture can be measured, including in the air, on material surfaces, and inside materials

Indoor moisture can be characterized with a number of parameters that are either directly measured *in-situ* or inferred from such measurements. Each parameter provides a different assessment of moisture depending on location, so there are not direct equivalencies between most of these parameters. This is important to consider when assessing the moisture of a specific building component (e.g. moisture at a gypsum drywall surface) because not all parameters will provide a representative characterization of moisture. The following sections describe the various moisture parameters that can be assessed in buildings. Figure 1 organizes the moisture parameters by measurement location, Fig. 2 illustrates the location of each measurement in an air/material system, and Additional file 1 provides a more detailed description of each parameter.

#### Moisture in the air

Moisture in the air is often assessed by measuring relative humidity (RH), which is a measurement of moisture saturation in the air and thus is a strong function of temperature. Air parameters that can be calculated from RH and temperature (or any two psychrometric parameters) include the humidity ratio (HR), defined as the ratio of the mass of water vapour to the mass of dry air; the water vapour partial pressure (*P*_vap_); and the vapour pressure balance (VPB), defined as the difference between indoor and outdoor vapour pressures, which is an indicator of indoor dampness [39]. Indoor RH and temperature vary spatially and temporally in buildings, and so measurement location and frequency can have a significant impact on the value of RH. HR, *P*_vap_, and VPB are temperature independent, so they are only affected by absolute changes in water vapour mass.

#### Moisture at a material surface

Aside from air, moisture can also be assessed at a material surface, ideally by measuring water activity (*a*_w_) since it is an intensive property that is unaffected by the volume or mass of the material being measured [40, 41]. However, *in-situ* measurements of *a*_w_ are currently impossible [9], largely because building surfaces are not at equilibrium. Instead, equilibrium relative humidity (ERH) is measured, as it is equivalent to *a*_w_ under equilibrium conditions and may be a reasonable approximation under small deviations from equilibrium. ERH is determined by measuring the RH in a sealed volume on a material surface (Fig. 2), which indicates the moisture in the air directly above the material surface. Similar to *a*_w_, ERH is a material-specific parameter because the RH in the sealed volume is a function of the moisture exchange between the air and the material. The main concerns with this approach pertain to the container used to seal the volume, as the size of the container affects the characteristics of the sealed air, and the container also alters the transfer of moisture and energy between the material and the surrounding environment.

#### Moisture within a material

If moisture within a material is of interest, a material’s moisture content (MC) can be determined *in-situ* by measuring an electrical property, which is typically either resistance or capacitance. The accuracy of these approaches is affected by a number of factors, including the presence of dissolved salts, electrical properties of the material, and alterations to material properties from inserted measurement probes (for resistance meters) or surface contact pads (for capacitance-based meters) [42]. There are also meter-specific considerations that affect measurements, and previous investigations have reported limits on accuracy and discrepancies between different measurement devices [8]. In general, assessing the MC is problematic because current measurement methods are somewhat empirical. All *in-situ* MC measurement techniques are essentially estimates of the MC value determined from a gravimetric approach, which is believed to yield the most accurate assessment of MC [43]. The gravimetric approach deduces MC from the difference in mass of a material specimen, caused by evaporation of moisture, before and after a period of oven drying [43]. There are potential errors associated with this approach as well (which are further discussed in Additional file 1), but the main issue for *in-situ* measurements is that gravimetric assessments are destructive and therefore not suitable for application in buildings. But regardless of the *in-situ* measurement approach and the entailed error, MC is problematic to assess because it is influenced by the bulk material being measured, there is no standard in situ measurement technique, and there is currently no available method to determine the MC of certain microenvironments (e.g. probes and contact pads do not provide a localised measurement of moisture at the specific area where fungi grow) [9, 40].

#### Additional moisture parameters

For all moisture parameters, an additional parameter, time of wetness (TOW), can be calculated. Traditionally applied to *a*_w_, TOW is the fraction of time that a given parameter is above a specific threshold [44]. It is useful because it indicates not only the magnitude of moisture, but also the duration of the magnitude. It can therefore identify potentially important events, such as periods of high moisture, which are likely relevant to fungal growth, which other parameters cannot. Given the differences between moisture parameters and hysteresis effects (further discussed in Section 2.2), there are likely important differences between the TOW for different parameters under the same conditions.

Although there are considerations with each of these measurements, they are useful for assessing moisture related to the three different locations. An additional layer of complexity is added when measuring the moisture associated with gypsum drywall in operating buildings and investigating how this can influence fungal growth. The purpose of the next section is to describe some of these complexities and the factors that influence moisture measurements. The effect of these parameters on fungal growth is discussed later in Section 2.3.

### Research question 2: how do building factors influence moisture parameters?

Measurement of any of the above moisture parameters is further complicated in buildings by a number of building design and operational factors. This section discusses how material properties, building assemblies, and indoor hygrothermal conditions influence the moisture parameters associated with gypsum drywall. The discussion pertains mainly to RH, ERH, and MC, since they are measureable *in-situ* parameters.

#### Effect of material properties

The moisture parameters associated with a building material are affected by its specific physical and chemical properties. Gypsum drywall is a complex, multi-layered building material system, typically consisting of a gypsum slurry core with a smooth finishing paper glued to the front side and a rougher paper covering glued to the back side [16]. The core of conventional interior drywall is composed of gypsum (i.e. calcium sulfate dehydrate, CaSO_4_ · 2H_2_O), starch, and small amounts of other additives [45, 46], while the paper coverings are composed mainly of cellulose and starch. Gypsum drywall’s density is reported to range from 600 to 1000 kg/m^3^ [47–50] and the boards come in a variety of thicknesses, ranging from a 0.25 to 1 in. [51]. Conventional gypsum drywall is hygroscopic and quite porous (e.g. specific areas ranging from 0.2 to 1.73 m^2^/g [52, 53], and porosities ranging from 0.305 to 0.65 m^3^/m^3^ [47, 54]) with buffering capabilities [3, 53] and a fairly high-water holding capacity [55] due to its internal pore structure [52]. The water vapour permeability of plain gypsum drywall ranges from around 23 ng/(Pa · s · m) at 10 % RH to about 45 ng/(Pa · s · m) [54], while its vapour diffusion resistance coefficient has been defined as 8.3 (dry cup, 3–50 % RH) and 7.3 (wet cup, 50–93 % RH) [50]. Based on these properties, it is not surprising that gypsum drywall is capable of holding almost its entire weight in water [8, 47, 54]. Moreover, gypsum drywall is very quick to absorb moisture but very slow to dry out [9]. This is due to the size, geometry, and distribution of pores in the gypsum core, as these affect vapour permeability and moisture diffusivity, as do the different layers. The paper coverings and gypsum core have different hygric properties, including water absorption capacity [55] and maximum MC [42], which will affect the moisture profile throughout the material (MC) and at the surface (ERH). Price and Ahearn [56] found that the finished paper side of drywall specimens had a higher MC than the back side, which illustrates this point.

Although this conventional interior type of drywall accounts for more than half of all drywall manufactured and sold in North America, there are many specialty boards that are used in different positions in building assemblies or to achieve different performance criteria. Fire-resistant drywall is made possible through the use of reinforced glass fibres and chemical additives. Water-resistant drywall usually incorporates reinforced glass fibres and fibreglass fabric coverings (or a similar coating) instead of conventional paper coverings to reduce its susceptibility to moisture (e.g. [34]). There are also less common products, including abuse-resistant drywall, which has a denser core and reinforced glass fibres; light-weight drywall, which has a higher porosity; and acoustic drywall, which incorporates viscoelastic sound-absorbing polymers. Conventional gypsum drywall is typically used for walls and ceilings, but ceiling drywall is also available, which is more sag-resistant and has a water-repellant interior finish. There are also boards available for wet-room application (e.g. bathrooms), which have a water-resistant core and water-repellant coverings. Aside from interior finishes, gypsum drywall is also used as an exterior sheathing material and in other exterior applications (e.g. the underside of exterior protrusions, such as soffits, canopies, and exterior insulation finishing systems). Exterior-use gypsum drywall typically consists of a water- and fire-resistant core, water-repellant paper coverings, and has a higher sag-resistance.

Gypsum drywall is typically installed as a single layer, but sometimes multiple applications are used if greater fire-resistance, strength, or sound-proofing is required. A single or base layer is attached to framing members mechanically with nails, screws, or staples, and a second layer can be attached mechanically or with a laminating adhesive. All joints and corners receive a finish layer treatment to hide the seams from parallel panels. Control joints can also be used for areas with a considerable amount of movement (e.g. long wall segments and wall-to-roof interfaces) and caulking is often used to seal edges to prevent the passage of air. The intended purpose of the drywall governs its position in a building assembly, which in turn, affects its moisture exposure.

#### Effect of building assemblies

The position of gypsum drywall in a building assembly can influence moisture diffusion and its associated moisture parameters. Installing gypsum drywall next to a material with a lower permeability can inhibit moisture diffusion from the drywall, thus increasing its MC [6], and potentially prolonging the TOW associated with MC. Drying via vapour diffusion can also be inhibited for interior gypsum drywall by finishing treatments, such as vinyl wallpaper or latex paint. For example, the water vapour permeability of gypsum drywall decreases from 32 ng/(Pa · s · m) at 50 % RH to 22 ng/(Pa · s · m) when one coat of primer is applied, and to 4 ng/(Pa · s · m) when one coat of primer and two coats of paint are applied [54]. Furthermore, many construction materials are/can be installed wet (e.g. wood and concrete) and so materials can become damp via moisture diffusion from adjacent materials when initially installed. It is recommended that the MC of the framing members that gypsum drywall is attached to be below 19 % [57]. If this is not adhered to, moisture could diffuse from a wood member to a neighbouring piece of drywall and increase its MC and ERH, as could contact with a cold component in a building assembly. An uninsulated chilled water pipe can cause water vapour in an air mass to condense if the pipe is below the dewpoint temperature of the air, which can increase the ERH and MC of an adjacent piece of drywall. Gypsum drywall is often in contact with thermal bridges (e.g. wall studs), which are highly conductive materials with a low thermal resistance. In cold outdoor conditions, there is an outflux of heat at these areas, which reduces the interior surface temperature and consequently increases the RH and ERH near the surface, thus increasing the potential for condensation [9].

The areas between thermal bridges often consist of insulating materials with different thermal and moisture properties in attempts to maintain a comfortable and energy-efficient indoor climate. This in turn creates the potential for dampness and condensation within a building assembly by creating a vapour pressure differential across the building assembly that causes moisture diffusion. Depending on geographic location, buildings can have both a heating and cooling season, which can lead to vapour diffusion from the inside or outside, depending on the outdoor and indoor conditions. Certain envelope features, such as a vapour barrier on the interior side, can lead to unwanted condensation and damp building materials (e.g. adjacent cavity insulation), which in this case, would likely be during the non-dominant cooling season in cold climates (e.g. [38]). Drying these moistened materials within a building assembly can be very slow processes that can last for years if the building assembly is not ventilated (e.g. through a vented cavity) or if finishing materials with low vapour permeabilities (e.g. vinyl wallpaper) are used. This scenario has been a classic and reoccurring problem for interior gypsum drywall (e.g. [38, 58]) in both residential and commercial buildings, as the inhibited drying potential has resulted in prolonged dampness and material damage [9]. The ability to dry is essential, as it is quite common for moisture to enter an assembly during both the operation and construction phase. During operation, events such as wind-driven rain and plumbing leaks can bring moisture into an assembly from both the inside and outside. During construction, humid outdoor conditions can entrap moist air and increase the MC of materials in the assembly. One study found that the MC of gypsum drywall could be around 8–10 % under very humid outdoor conditions during construction [8], which can lead to material degradation. After construction, buildings are conditioned for occupant comfort, and the intent is that indoor moisture is better controlled; however, this is not always realized in a sufficient manner to prevent microbial growth.

#### Relationship among parameters under transient indoor hygrothermal conditions

Even in a well-designed building, indoor temperature and RH vary spatially and temporally. Geography, seasonality, and outdoor weather conditions influence indoor temperature and RH in a region, while building-specific factors such as ventilation, occupancy, and building type create smaller-scale differences among and within buildings. These indoor climate dynamics cause moisture flows within and between materials and air in a building, as well as changes in the various moisture parameters [9]. The ERH of gypsum drywall can increase in environments with either high or low RH. When ambient RH is low, there is a moisture gradient that decreases from material to air, and so pore water in a piece of gypsum drywall will be driven to the surface and increase ERH while decreasing MC [59]. On the other hand, when RH increases, water from the air will be adsorbed to the drywall surface due to gypsum drywall’s hygroscopicity, and consequently increase ERH, and also MC if sufficient moisture absorbs into the pore structure [60]. This in turn implies a high ERH TOW since gypsum drywall’s surface can remain moist in high or low ambient humidity (and is further influenced by gypsum drywall’s quick absorption and slow desorption rates) [9]. MC is also affected by ambient RH, and as explained above, the two parameters typically fluctuate in the same direction, except at high RH values close to saturation, where the relationship is often variable [9]. However, MC is not a direct function of RH and so it cannot be determined from an RH measurement. The MC of gypsum drywall is affected differently depending on whether RH is increasing or decreasing. In general, the MC will be higher during periods of increasing RH than it would be during periods of decreasing RH, which is typically illustrated via sorption isotherms [44]. This is caused by the hygric properties (i.e. quick absorption and slow desorption) of gypsum drywall and the fact that moisture diffuses through air at a much faster rate than it does through materials [61]. This latter point also explains why there are variations in the difference between air humidity (RH) and surface humidity (ERH), and also why the ERH of gypsum drywall was found to be most similar to air RH during periods of increasing RH, and least similar during periods of decreasing RH [36]. Furthermore, there is not always a direct relationship between gypsum drywall’s MC and ERH. Although an increase in MC can lead to an increase in ERH, van Laarhoven and colleagues found that gypsum drywall specimens with the same surface moisture (*a*_w_) could have very different MCs, depending on whether the sample had been exposed to high air RH (i.e. water vapour) or an aqueous solution (i.e. liquid water) [59]. Indoor moisture is therefore an important indoor environmental consideration, as it is highly dynamic and affects the moisture parameters associated with a material in distinct ways.

### Research question 3: what impact does moisture have on fungal growth?

When indoor moisture is not properly managed, the moisture parameters associated with a material can reach high levels, which can lead to bacterial and fungal growth. Fungi require adequate temperature, nutrients, and moisture to grow. In buildings, moisture is believed to be the only limiting factor for fungal growth, as the temperature range that buildings are conditioned to (for occupant comfort) falls within the wide range of temperatures that fungi can grow in, and substrate nutrient requirements are satisfied by the constituents of common building materials [44, 47, 62]. Conventional gypsum drywall contains starch, cellulose, and adhesives, which provide an abundance of nutrients for fungi (e.g. [38, 45]). Specialty gypsum boards, such as moisture-resistant boards that incorporate fibreglass fabric coverings instead of paper, often contain fewer nutrients than conventional boards, but will still provide sufficient nutrients for growth [34], due in part to a layer of dust, microorganisms, and organic materials, that is acquired from the surrounding environment [62]. It is not surprising then, that both laboratory and field studies observed fungal growth on gypsum drywall samples that had been exposed to some form of excess moisture [27, 61, 63, 64]. The most common genera these researchers found were *Stachybotrys* [15, 32, 65–67], *Chaetomium* [67], *Aspergillus* [37, 66, 68], *Penicillium* [15, 37, 68], and *Ulocladium* [32, 67], which is not surprising as these genera are common indoor fungi (e.g. *Aspergillus* and *Penicillium*) and favour starch and cellulose for digestion (e.g. *Stachybotrys* and *Chaetomium*). Other genera, such as *Cladosporium*, *Acremonium*, *Mucor*, *Paelomyces*, *Alternaria*, and *Verticillium*, have also been observed on gypsum drywall, but much less frequently [67].

#### Moisture measurement in microbial investigations

**Fig. 3:**
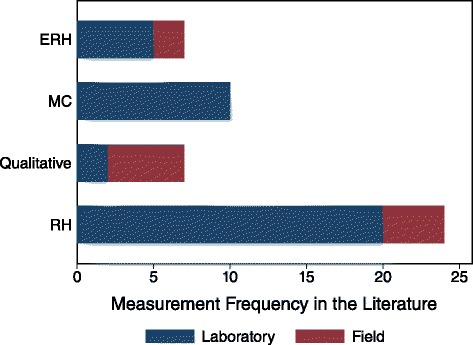
Frequency of measured moisture parameters from original research in the literature. Quantitative moisture parameters for air (RH), surfaces (ERH), and materials (MC), as well as qualitative assessments of moisture were recorded from the sources that were surveyed

Despite these cases and the known importance of moisture to microbial growth, there is no consistent approach used to assess moisture in the literature. To investigate this matter, a literature review was conducted on sources that included an assessment of both moisture and microorganisms associated with gypsum drywall, including peer reviewed research articles, building standards, construction guidelines, and reports. The sources were first analysed to establish how moisture has been measured in previous experimental studies that investigated moisture-induced microbial proliferation on gypsum drywall in either a laboratory or field environment. Only sources that involved an investigation of moisture and microbial communities related to gypsum drywall and a first-hand moisture measurement were included (i.e. sources that addressed moisture but did not make a measurement, such as building standards, were not included in this first analysis). The results of this first review are summarized in Fig. 3 and Additional file 2, which show the measurement frequency of each moisture parameter, sub-divided by measurement environment (i.e. laboratory vs. field). Additional information for Fig. 3, including reference information, study environment, and the moisture parameter measured, is provided in Additional file 2. Air RH, surface ERH, material MC, and qualitative observations of moisture made during the experiments were the only parameters recorded from the literature (preliminary assessments of material properties that involved moisture were not recorded). Of the 28 references that met this review criterion, 14 studies measured more than one moisture parameter, while the other 14 assessed only one. Moisture measurements were made in laboratory environments only in 19 studies, while 5 studies assessed moisture in the field only, and the remaining 4 studies assessed moisture in both environment types. RH is the most common measurement taken in laboratory studies and overall, with only 6 of 28 studies not including any assessment of RH. The measurement frequency of the other three parameters was fairly equal overall, with qualitative observations being made mostly in field investigations (which is likely due to the ease of measurement in this environment) and MC measurements being made mainly in laboratory studies. In general, it is evident that there is no consistent moisture parameter measured in either type of the study, which makes interpretation of moisture from the literature and comparison among studies difficult because different types (i.e. air, surface, and material) of indoor moisture are characterized in different ways.

#### Critical moisture levels for microbial growth

**Fig. 4:**
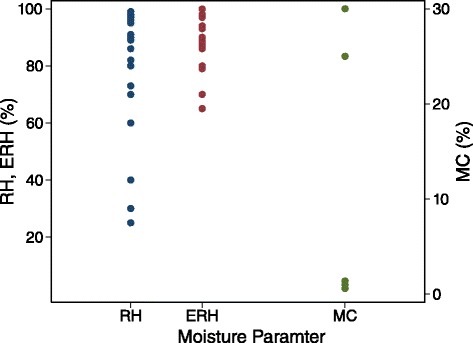
Critical moisture values to prevent fungal growth on gypsum drywall from the literature

Another implication of this discrepancy is disagreement pertaining to the moisture conditions that cause fungal growth. Several studies define a critical moisture value below which, fungal growth will not occur. However, since there is no consistent type of indoor moisture assessed in the literature (Fig. 3), it is difficult to establish a unanimous moisture threshold. To investigate this matter, a second analysis of the same references described above was conducted whereby critical moisture values were extracted from each source that explicitly stated one. Values specified for gypsum drywall were recorded, along with those for indoor environments and surfaces in general. Critical moisture values were recorded from 28 sources and are presented in Fig. 4 and Additional file 3, categorized by parameter (i.e. air RH, surface ERH, and material MC). Forty-three, 29, and 5 critical values were recorded for RH, ERH, and MC, respectively, with several studies defining more than one critical value based on different experimental conditions (e.g. temperature). These threshold values span the largest range for RH, followed by ERH and MC, which both encompass a similar range of values. Although the range is largest for RH, the coefficient of variation is largest for MC, which is surprising considering the fact that although gypsum drywall’s MC can reach high values [8, 69], it typically remains below 1 % in indoor environments. Greenwell and Menetrez found the MC of gypsum drywall to be 0.3 % when exposed to typical indoor conditions of around 20 °C and 50 % RH [69], while Laurenzi observed MC values of gypsum drywall to range from 0.7 to 0.8 % when exposed to more extreme RH levels close to saturation (i.e. 90–95 %) [70]. The large range of critical MC values observed in this review could be a result of different MC measurement approaches, which include both gravimetric and electrical-based measurements in the analysed studies, with the two highest values being measured on specimens non-intrusively through a plastic freezer bag [56]. Researchers have noted differences between gravimetric and resistance-based MC readings on gypsum drywall specimens [8] and MC is known to exhibit considerable spatial variation, even over a few centimeters, [71], which could also help explain the large difference in magnitude between these two higher values and the other three.

**Fig. 5:**
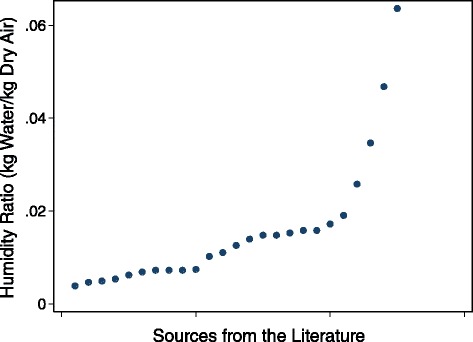
Critical moisture values to prevent fungal growth on gypsum drywall from the literature, expressed as HR values using stated RH and temperature values

In general, the variation in defined critical moisture values within a specific moisture parameter can be attributed to variations in measurement scenarios and possible sources of error associated with different moisture measurement techniques. However, the main cause is likely due to the measurement location (i.e. air, surface, and material) and its relevance to microbial growth. In buildings, microbial growth occurs at a material. For gypsum drywall, this typically happens at the surface [24, 43] or at an interfacial layer, specifically on a starchy component [24], such as the underside of the drywall’s paper covering. Although the core can support fungal growth on its own [45, 46], drywall in buildings almost always includes a covering material, which likely explains why growth typically starts at the paper liner and sometimes propagates into the core [24]. Surfaces and interfacial layers are most often the site of growth because moisture is more commonly available at these locations. This explains why the critical moisture values defined by ERH exhibit the least amount of variation, and also why several others have identified surface moisture (defined as either ERH, *a*_w_, or liquid water on a material surface) as most relevant to microbial growth [16, 24, 44, 62, 72]. Since ERH is a measure of available moisture at a material surface, high values of ERH could lead to surface growth and/or a moistened paper covering, which could result in growth on the back side of the finishing paper. Although MC is a measure of moisture within a material, the threshold values based on MC exhibit a considerable degree of variation because water within a material is not necessarily available for fungi, as it is often bound within the pore structure. Although a high MC could lead to microbial growth if the paper covering becomes damp, or if pore water desorbs to the surface (due to a decrease in RH), it is not guaranteed, as the internal water might be bound within the material. Lastly, the critical values defined by RH encompass a large range because RH is an assessment of moisture in the air, which is not necessarily available for fungal growth, unless it is absorbed into the material or condenses at the surface. Surface condensation can occur at a range of air RH values (even when the surrounding RH is below saturation) because condensation can occur in pores due to a build-up of absorbed moisture, and also if the surface temperature is below the dewpoint temperature of the ambient air [9]. Since RH measurements related to fungal growth are essentially assessments of condensation potential, HR could be a more useful air measurement since it defines the absolute amount of moisture in air, which is unaffected by temperature variations. To investigate this, HR threshold values were calculated for the RH threshold values shown in Fig. 4 and Additional file 3 where temperature data was available, and are presented in Fig. 5.

Figure 5 shows variation in the critical moisture values defined by HR and a large range overall. In North America, HR typically varies between 0.001 and 0.02 outdoors [39] and between 0.0075 and 0.0098 in residential buildings [73], so it is evident that many of the values in Fig. 5 are unlikely to occur in most buildings. Yet, indoor moisture problems are commonplace and it has been reported in a number of surveys that at least 20 % of buildings have experienced some type of moisture problem [11]. This implies that indoor HR might not be the most appropriate indicator of a moisture problem, which is likely due to complexities, such as measuring indoor temperature and understanding material-specific condensation conditions, and the fact that moisture in the air is not necessarily indicative of localised areas of high moisture (e.g., at a material). Indoor temperature, which is required to determine HR from a RH measurement, varies spatially due to the different thermal capacities of materials and vertical stratification. This leads to immense variation within an individual room, making it difficult to determine a representative value for indoor temperature, which in turn, influences the interpreted value of HR. Another difficulty in defining a critical moisture value with HR pertains to the fact that the air humidity required for capillary condensation for a specific material is dependent on its pore structure and can be much lower than 100 % RH [9]. Surface temperatures can be much lower than that of the ambient air and fall below the dewpoint temperature. In this scenario, surface condensation can occur even if ambient moisture levels seem much lower than saturation. Finally, the amount of moisture in the air might not reflect a smaller area of high moisture at a surface that could lead to fungal growth. This explains why other experts have recommended monitoring dewpoint temperatures [58] as opposed to ambient humidity, and why the use of either air parameter (i.e. RH and HR) to define a consistent critical moisture value has been unsuccessful in this review [62, 64, 74].

These findings may suggest that fungal growth on gypsum drywall is present largely as a function of surface moisture, particularly liquid water, rather than from the presence of water vapour in the air. This helps to explain why surface wetting has been observed to have a prominent influence on microbial proliferation, despite the fact that it provides no quantitative assessment of moisture and can be performed in a number of ways (e.g. submersion, dripping, etc.). Doll and Burge [16] found that increasing moisture in a manner that mimicked a wetting event led to increased fungal growth. They also found fungal growth on gypsum drywall specimens that were exposed to a high RH to be minimal if they had not been subjected to a wetting event [16]. Similarly, Horner and colleagues [72] found that fungi grew on gypsum drywall samples under a low RH (even at 0 % RH) if they had previously been wetted, and Hoang and colleagues [75] noted a distinct difference in microbial growth rates on naturally inoculated “green” and conventional gypsum drywall samples depending on whether they experienced a wetting event or exposure to high RH. Furthermore, van Laarhoven and colleagues [59] found that growth rates on gypsum drywall samples with the same surface moisture (*a*_w_) were faster if they had been submerged in an aqueous solution (which mimics a wetting event) rather than exposed to a high ambient RH. More generally, ASHRAE states that “the factors that lead to microbial contamination, including mold, are catastrophic water damage, repeated wetting, or excessive long-term moisture accumulation in materials” and that building materials should be kept as dry as possible to prevent microbial proliferation [71]. Harriman and Brennan [58] further stress this point, by stating that it is liquid water and not high ambient humidity that poses the greatest threat. These findings, along with the large variation exhibited by critical moisture values defined by air RH and HR, imply that indoor air moisture might not be directly important to fungal growth. This is likely to be the case in buildings, as the ambient RH does not provide information about the moisture in the very small layer of air at a material surface, which is likely to have a different thermal capacity (and therefore temperature) [71] and buffering capability than the ambient air.

#### Identifying critical moisture

Although surface moisture, including ERH and surface wetting, appear to be most indicative of fungal growth, defining a single critical moisture value to prevent fungal growth on gypsum drywall is still difficult because fungal growth is variable depending on a number of factors aside from moisture, including fungal taxa, temperature, and substrate characteristics. First, different fungal species have different tolerances to moisture; some will grow at lower moisture levels (e.g. primary colonizers will grow at ERHs below 80 %), while others will only grow at high moisture levels (e.g. tertiary colonizers will grow at ERHs above 90 %) [76]. Doll and Burge [16] and Pasanen and colleagues [64] have observed this in laboratory studies where a gradual emergence of primary, secondary, and tertiary colonizers on gypsum drywall samples occurred with increasing moisture levels. Second, fungi can grow and survive in a wide range of temperatures; however, every species has a specific optimum temperature for growth [9, 33, 62, 76]. The temperature range that buildings are conditioned to is unlikely to inhibit growth [2]; however, it can slow growth rates as temperatures diverge from optimum. When this happens, surface moisture requirements increase [2], so in other words, the critical ERH for a fungus to grow is lowest at its optimum temperature and increases as temperature diverges [9]. Third, material constituents and properties have been found to affect fungal growth rates and the species that proliferate. Adan [44] states that a reduction in material porosity will result in a reduction of fungal growth, while others have observed differences in growth among different types of drywall [8] and also differences on the front, back, and sides of the individual samples of gypsum drywall [16, 75]. Murtoniemi and colleagues have demonstrated that fungal growth varies among different types of gypsum drywall [45, 65]. Biomass production and sporulation decreased substantially on gypsum drywall samples that were desulphurized, contained less starch, or had been treated with a biocidal substance [46], whereas removing only a single additive and incorporating a core made of recycled boards did not have a significant effect. Interestingly, *Penicillium* grew almost exclusively on the gypsum cores [45], and when the liners and core were treated with biocides separately, growth still occurred on the core, but was almost completely inhibited on the liner [45]. Another complication with substrate materials is that they age and degrade over time, and also acquire a surface coating of dust and other organic matter, which has been found to affect material properties (e.g. vapour permeability, among others) and growth rates [5]. Surface treatments and coatings can be altered throughout a building’s lifetime, and this has been shown to affect growth [63], especially if a vapour-retarding finishing product, such as an oil-based paint, is applied [58, 60]. Lastly, microbial communities growing on a material can alter the material’s properties and govern the moisture conditions for continued growth. Onysko and colleagues [77] found that microbial growth can alter the vapour permeability of a material, and Adan [44] found that *Penicillium chrysogenum* can intake water the instant RH increases, which shows that the substrate’s hygric properties had less of an effect on the fungus’ growth once it had commenced. Overall, the literature explains the many dynamic factors that influence microbial growth, and also provides a number of examples where growth has occurred under different environmental conditions. The different growth requirements of different fungi, as well as variations in indoor conditions and material properties over time create the potential for microbial growth under various circumstances, and further explain why a single critical moisture value cannot be defined.

An alternative approach to defining a critical moisture value would be one that follows the underlying methodology of the “lowest isopleth for mold” (LIM) model [47, 78, 79], which recognizes that the conditions for mold germination and growth differ depending on fungal species and addresses this by developing isopleth systems of temperature and RH for specific fungi on specific substrates (including optimum growth media and different types of building materials) using measured data [78, 79]. The lowest line of temperature and RH is deemed the LIM, which is a more dynamic indicator of fungal growth than a critical moisture value. This dynamic analysis is made available for real buildings through computational modelling programs, such as WUFI 2D and WUFI Bio. These programs utilize a biohygrothermal model, which incorporates the LIM system and isopleths for mold growth on specific materials, as well as transient climatic conditions (i.e. temperature and RH), to determine the water balance within fungal spores in order to estimate the potential for mold growth [47, 80, 81]. WUFI 2D uses this approach to estimate whether or not mold will germinate and grow, and WUFI Bio incorporates an additional layer by comparing environmental situations to other growth curves in order to predict the level of growth/infestation [81]. These computational tools have been utilized in many studies (e.g. [82]) and are continuously validated against measured data [83]. However, external comparison between measured and modelled data reveals that WUFI Bio might not provide accurate estimates of mold growth in all scenarios, as one study found general disagreement between measured and modelled results (using WUFI Bio) of mold growth in UK dwellings [84]. Currently, these models might not provide accurate predictions for all scenarios and also cannot yet model the specific stages and details of growth or anticipate all scenarios during a building’s life. Further validation would enhance the predictive accuracy of these programs, which have the potential to provide useful predictions of possible moisture and mold problems in buildings under certain scenarios, so long as the user is cognizant of the current limitations.

Aside from computational modelling, *in-situ* moisture measurements can identify unanticipated moisture and mold problems, so long the right approach is used. Available moisture at a material surface has been identified as the most influential parameter [55, 63], and so surface measurements of ERH and the associated TOW are likely the most appropriate measurements [44, 52, 55, 62, 63]. However, these parameters vary depending on location in a building, so special consideration of the measurement area should be given. Moisture-prone and colder areas (e.g. typically thermal bridges, envelope penetrations, and interfaces in a building structure) would be important areas to assess since they are more likely to be sites of condensation and high surface moisture. A more thorough approach would be to conduct an initial building moisture audit. “Musty” odors are indicative of dampness [59] and an infrared (IR) device can identify specific areas of excess moisture that could be important to monitor. Once an area is identified, assessing surface TOW would be especially useful because it quantifies the magnitude of surface wetness as well as the duration of wetness [44]. This is particularly important when measuring the surface moisture associated with gypsum drywall because this material is very quick to get wet, but very slow to dry out (due to its hygroscopic properties and pore structure), which means that even a short period of very high ERH can entail an extended period of surface dampness, which could lead to fungal growth. Measurements should be long term and continuous because indoor hygrothermal conditions are dynamic, and a single measurement will not provide a comprehensive assessment of the range and fluctuations that surface moisture encompasses. Despite the lack of a single critical moisture value to prevent fungal growth on gypsum drywall, this measurement approach will provide continuous information on surface moisture at a specific area, which could be telling of the various scenarios that could lead to indoor fungal proliferation.

#### Summary recommendations

The findings from this review entail several recommendations for both researchers and practitioners. The recommendations for researchers are as follows:

- Different types of gypsum drywall had a significant effect on fungal sporulation and growth, yet the physical and chemical properties of gypsum drywall, including both the core and covers, are seldom characterized in the literature [45, 65]. Accordingly, future investigations should include much better characterizations of the materials used in experiments.
- Different relationships between moisture parameters have been observed under similar moisture levels (e.g. different MC values at the same *a*_w_, depending on how the specimen was conditioned) [59]. Further research should be conducted on the interactions among different moisture parameters under transient indoor hygrothermal conditions.
- As new products are developed, their properties and resistance to mold growth should be tested. Murtoniemi and colleagues [45, 46] have noted different growth rates on different types of gypsum drywall. Growth rates on new materials should be investigated before materials are used in buildings.
- The use of different microbial methods has been shown to yield different characterizations of microbial communities [30]. Further efforts need to be put towards making a standard and verifiable approach.

For practitioners, the recommendations include:

- More diligent monitoring of buildings by occupants and owners in order to identify musty odors and areas of visible moisture damage, which could indicate a moisture problem [58].
- Engineers, designers, and building owners should be more cognizant of the more appropriate ways to investigate a moisture problem (i.e. *in-situ* surface moisture measurements, computational predictive modelling, IR inspections), as well as the various available guidelines and standards that provide advice on controlling moisture. Building codes should be updated to reflect these best practice approaches.
- Practitioners should record and share findings from real-building investigations with researchers, to help translate results from the laboratory to real buildings.

These recommendations are intended to improve researcher and practitioner understanding of moisture-induced fungal growth on gypsum drywall in buildings, and also highlight appropriate measures to identify and determine moisture levels in buildings that could lead to fungal growth.

## Conclusions

The literature consists of many papers that investigate moisture and fungal communities associated with gypsum drywall. However, the collective results do not lead to consensus on the three research questions. First, there are various in situ moisture parameters that can be measured or inferred to characterize moisture associated with the three locations in buildings, which include moisture in the air, at a material surface, and within a material. A review of moisture measurements made in studies that examined moisture and fungal growth on gypsum drywall reveals no consistent parameter measured in laboratory and field studies. RH was identified as the most common measurement in laboratory settings (and overall), and qualitative observations of moisture were most common in field investigations. Second, although several papers provide a thorough description of moisture dynamics in buildings, indoor conditions are transient and difficult to control, and accordingly, indoor fungal growth is difficult to predict. A review of critical moisture values to prevent fungal growth from the literature shows substantial variation for values defined by RH and MC, and less variation for those values defined by ERH. A primary cause for these variations is that moisture requirements for fungal growth vary based on fungal species, temperature, and nutrients, so it is difficult to define a single moisture threshold. Another important factor to consider is the relevance of the moisture measurement to fungal proliferation. Available moisture at a material surface has been identified as most relevant to fungal growth, and so surface measurements of ERH and surface TOW are useful for monitoring specific, localised areas of buildings, and will provide the best indication of scenarios that lead to fungal growth. Long-term, continuous monitoring is the best *in-situ* approach to characterize indoor moisture, as it will capture the range of values a moisture parameter encompasses in response to spatial and temporal variations in indoor hygrothermal conditions. Although a number of uncontrolled, transient indoor environmental factors make moisture and microbial growth difficult to predict in buildings, this measurement approach can provide insight on the numerous scenarios that could lead to moisture-induced fungal growth on gypsum drywall in buildings.

The three additional files are intended to provide supporting information on the various moisture measurement parameters discussed in this paper, as well as information on the sources used in this review. Additional file 1 provides a more detailed explanation of each measurement parameter discussed in “Research Question 1”, along with a more extensive discussion of measurement challenges and considerations. Additional file 2 presents a classification of the different moisture parameters measured in field and laboratory studies in the literature that are presented in Fig. 3. Lastly, Additional file 3 lists the numeric moisture threshold values that are presented in Fig. 4, along with information pertaining to the specific scenario that the value applies to. This file also includes the temperature data (where available) that was used to calculate the threshold HR values shown in Fig. 5.

## Additional files

Additional file 1: Table S1. Additional information for the moisture parameters defined in Section 2 of the main text. This table provides additional information on the various measurable and inferable moisture parameters discussed in Section 1. This includes a more detailed explanation of the parameters and a more extensive discussion of considerations and challenges with each approach [85, 86]. (DOCX 29 kb) Additional file 2: Table S2. Moisture measurement frequency in the literature—additional information for Fig. 3 in the main text. This table presents the moisture parameters measured, categorized by measurement environment, in the 27 studies data used to create Fig. 3 [87–90, 92–94]. (DOCX 23 kb) Additional file 3: Table S3. Additional information for critical moisture values to prevent microbial growth defined in the literature. Corresponds to Figs. 4 and 5 in the main text. This table presents the critical moisture values extracted from the literature that were used to generate Figs. 4 and 5, and includes information on temperature, environment, document type, and scenario [95, 91, 96–98]. (DOCX 34 kb)

## Abbreviations

a_w_: water activity
ERH: equilibrium relative humidity
HR: humidity ratio
LIM: lowest isopleth for mold
MC: moisture content
P_VAP_: water vapour pressure
RH: relative humidity
TOW: time of wetness
VPB: vapour pressure balance
