# Supplementary material for: Moisture parameters and fungal communities associated with gypsum drywall in buildings
Source: Microbiome. 2015 Dec 8;3:71. doi: 10.1186/s40168-015-0137-y (PMC4672539; doi:10.1186/s40168-015-0137-y)
Supplement: Additional file 1: Table S1. — Additional information for the moisture parameters defined in Section 2 of the main text. This table provides additional information on the various measurable and inferable moisture parameters discussed in Section 1. This includes a more detailed explanation of the parameters and a more extensive discussion of considerations and challenges with each approach [85, 86]. (DOCX 29 kb) [file 40168_2015_137_MOESM1_ESM.docx]

Table S1: Additional Information for the Moisture Parameters Defined in section 2 of the Main Text

| **Medium** | **Parameter** | **Definition** | **Mathematical Formulation** | **Measurement Considerations** |  |
| --- | --- | --- | --- | --- | --- |
| Air | Relative Humidity  (RH) | -A measure of moisture in a volume of air. | $RH= \frac{P_{vap}}{P_{vap sat}}*100\%$ | -Temperature-dependent. |  |
|  |  | -The ratio of the vapour pressure of water in the air to the vapour pressure of water in air at saturated conditions at the same temperature and pressure [42]. |  | -Location is important, as RH can vary within buildings due to spatial variation in ambient hygrothermal conditions. |  |
|  |  |  |  | -Measurement frequency is important, as RH varies temporally, so continuous monitoring is required to determine the range of RH. |  |
|  | Humidity Ratio  (HR) | -Absolute mass of moisture in a mass of air. | $HR= \frac{M_{water}}{M_{dry air}}$ | -Temperature-independent, but can be calculated from measured values of any two psychrometric variables (e.g., temperature and RH). |  |
|  |  | -Ratio of the mass of water vapour in a volume of air to the dry mass of that volume of air [42]. |  | -Measurement location and frequency are important as these influence the inferred value of HR. Continuous measurements with special attention to the location of temperature measurement is recommended. |  |
|  | Vapour Pressure  ($P_{vap}$) | -The partial pressure of water vapour in air. |  | -Temperature-independent, but can be calculated from measured values of any two psychrometric variables (e.g., temperature and RH) or HR directly. |  |
|  | Vapour Pressure Balance  (VPB) | -The difference between indoor and outdoor vapour pressure. | $VPB= P_{vap indoor}-P_{vap oudoor}$ | -Temperature-independent because the molar concentration of water in air is temperature independent [39] |  |
|  |  | -A positive VPB indicates a damp building and a negative VPB indicates drier conditions [85]. |  | -Can still be influenced by measured values of temperature and RH, if inferred from these. |  |
|  |  |  |  | -Long-term continuous measurements of temperature and RH both inside and outside are required, and the location of these measurements is important due to spatial and temporal variations of these parameters. |  |
|  |  |  |  | -Difficulties have been encountered when interpreting values at outdoor temperatures above 20 °C, as the use of air conditioning can cause dehumidification that affects the perceived indoor moisture generation [39]. |  |
|  |  |  |  | -Changes in outdoor weather, such as precipitation events, can create very damp outdoor conditions that also skew the vapour pressure balance [39]. |  |
|  |  |  |  | -Provides an overall estimate of indoor dampness only, and does not provide an indication of localised moisture problems. |  |
| Surface | Water Activity (a_w_) | -Ratio of water vapour pressure in a material to the vapour pressure of pure water at the same temperature. | $a_{w}=\frac{P_{vap material}}{P_{vap pure water}}$ | -Advantageous because it is an intensive property that is unaffected by the volume or mass of the material being measured. It is however, affected by dissolved salts [9]. |  |
|  |  | -Describes the energy status of water in a system, which gives an idea of the escaping potential of water from the material, and therefore the water availability at a material surface [42]. |  | -*In-situ* measurements are currently impossible because materials are not in equilibrium in dynamic indoor environments. |  |
|  | Equilibrium Relative Humidity  (ERH) | -In-situ surrogate for a_w_ under equilibrium conditions only. | $ERH= {RH}_{in a sealed volume}$ | -Measured in buildings by sealing a small volume on the material of interest, and measuring the RH in the sealed volume once the material and sealed air have reached equilibrium (i.e., a net zero transfer of moisture across the air-material interface). |  |
|  |  | -Describes the moisture in the air directly above a material surface. |  | -Sealing the volume alters the material properties and can potentially impede the transfer of moisture and energy between the sealed volume and the surrounding environment. |  |
|  |  |  |  | -The geometry and material of the container used to seal the space will alter the air conditions inside the container. |  |
|  |  |  |  | -ERH varies along an individual surface. |  |
|  |  |  |  | -An individual sensor measures a very small space. |  |
|  | IR Techniques | -Identify surface temperature depressions caused by evaporation of moisture. | None | -Qualitative only. |  |
|  |  |  |  | -Provides an overall visual assessment of moisture. |  |
|  |  |  |  | -Good for assessing large areas. |  |
|  |  |  |  | -Time-sensitive (e.g., a moisture event might not be detected if it was a single-wetting event and evaporation has occurred prior to the IR investigation). |  |
|  |  |  |  | -Provides surface-level detection only (e.g., a moisture problem behind a surface will not be detected if it does not alter the surface temperature). |  |
| Material | Moisture Content  (MC) | -Quantitative measure of liquid water in a material [9]. | $MC= \frac{M_{water}}{M_{material}}$  $MC= \frac{V_{water}}{V_{material}}$ | -Dissolved salts affect resistance meter readings. |  |
|  |  | -Volumetric or mass ratio of water to the bulk material. |  | -Resistance meters are calibrated for a specific material, and so they might provide different readings for different types of drywall. |  |
|  |  | -For *in-situ* measurements, MC is inferred from an electrical property (typically either electrical resistance or dielectric permittivity). |  | -Resistance meters provide a measurement at a specific location and depth (not a value for the entire material). |  |
|  |  | -Resistance meters require probes be inserted into the material. |  | -Resistance meter probes should be insulated. If uninsulated, contact with liquid water can skew the measurements. |  |
|  |  | -Dielectric meters use surface contact pads. |  | -Dielectric meters are affected by a material's relative density (specific gravity) and contact with adjacent materials) [42]. |  |
|  |  | -Various other techniques exist for determining MC in a laboratory, with gravimetric assessments being most common. |  | -Electrodes (i.e., resistance meters) and surface contact pads (i.e., dielectric meters) can alter a material's hygric properties. |  |
|  |  | -The gravimetric approach involves weighing a specimen before and after oven-drying. MC is then determined from the difference in weight (caused by evaporative losses). |  | -The gravimetric method is not desirable for *in-situ* measurements, as it destructive [86]. |  |
|  |  |  |  | -Oven-drying, used in the gravimetric method, can skew the observed value. An excessively high temperature can cause excessive drying (i.e., not only water, but also material constituents could be driven off) and low temperatures might not evaporate all contained water (i.e., if equilibrium is reached in the oven) [43]. |  |
|  |  |  |  | -MC is an empirical measurement with no consistent measurement approach when determining the MC in a fungus’ microenvironment [9]. |  |
|  |  |  |  | -MC is affected by the bulk material and influenced by its properties (e.g., capillary pressure and pore characteristics) [9]. |  |
|  |  |  |  | -Discrepancies among different MC measurement devices [8]. |  |
| All Media | Time of Wetness (TOW) | -The amount of time that a moisture parameter is above a certain threshold. | $TOW= \frac{T_{>threshold}}{T_{total}}$ | -Many researchers have used TOW in relation to surface moisture with a threshold of 80% [e.g., 44], but any threshold can be specified and it can be applied to any quantitative moisture parameter. |  |
|  |  | - Typically expressed as a fraction of the amount of time above the specified threshold, to the total time in the monitoring period [e.g., 44]. |  | -Useful because it accounts for the magnitude of moisture and the duration of the magnitude, which provides insight on certain events of interest that other parameters might not capture (e.g., short periods of high moisture) and allow for comparison among moisture parameters, highlighting differences among these parameters caused by indoor moisture dynamics. |  |

*M* = mass

*V* = volume

*T* = time
