# Supplementary material for: Moisture parameters and fungal communities associated with gypsum drywall in buildings
Source: Microbiome. 2015 Dec 8;3:71. doi: 10.1186/s40168-015-0137-y (PMC4672539; doi:10.1186/s40168-015-0137-y)
Supplement: Additional file 2: Table S2. — Moisture measurement frequency in the literature—additional information for Fig. 3 in the main text. This table presents the moisture parameters measured, categorized by measurement environment, in the 27 studies data used to create Fig. 3 [87–90, 92–94]. (DOCX 23 kb) [file 40168_2015_137_MOESM2_ESM.docx]

Table S2: Moisture Measurement Frequency in the Literature – Additional Information for Figure 3 in the Main Text.

| **Source** | **RH** | | **ERH*** | | **MC** | | **Qualitative** | |
| --- | --- | --- | --- | --- | --- | --- | --- | --- |
|  | **Lab** | **Field** | **Lab** | **Field** | **Lab** | **Field** | **Lab** | **Field** |
| [44] | X |  | X |  | X |  |  |  |
| [27] |  |  |  |  |  |  |  | X |
| [87]^1^ | X |  |  |  |  |  |  |  |
| [4] | X |  |  |  | X |  |  |  |
| [8] | X |  |  |  | X |  |  |  |
| [88]^2^ |  |  |  |  | X |  |  |  |
| [16] | X |  |  |  | X |  |  |  |
| [76] |  | X | X | X |  |  |  |  |
| [75] | X |  |  |  | X |  |  |  |
| [72] | X |  |  |  |  |  |  |  |
| [15] |  |  |  |  |  |  | X | X |
| [25] | X |  |  |  |  |  |  |  |
| [89 |  | X |  |  |  |  |  |  |
| [63] | X |  |  |  |  |  |  |  |
| [14] | X |  |  |  |  |  |  |  |
| [23] | X |  |  |  | X |  |  |  |
| [63] |  |  | X |  |  |  |  | X |
| [90] | X |  |  |  |  |  |  |  |
| [24] | X |  |  |  |  |  |  |  |
| [74] | X |  |  |  |  |  |  |  |
| [64] | X | X |  |  |  |  |  | X |
| [55] | X |  | X |  | X |  |  |  |
| [456] | X |  |  |  |  |  | X |  |
| [92] | X |  |  |  | X |  |  |  |
| [93] |  |  |  |  |  |  |  |  |
| [29] |  |  |  |  |  |  |  | X |
| [36] |  | X |  | X |  |  |  |  |
| [59] | X |  | X |  | X |  |  |  |

*Measurements of a_w_ have been categorized under ERH because the two are equivalent under equilibrium conditions.

^1^ As cited in [9].

^2^ As cited in [94].
