# Supplementary material for: Moisture parameters and fungal communities associated with gypsum drywall in buildings
Source: Microbiome. 2015 Dec 8;3:71. doi: 10.1186/s40168-015-0137-y (PMC4672539; doi:10.1186/s40168-015-0137-y)
Supplement: Additional file 3: Table S3. — Additional information for critical moisture values to prevent microbial growth defined in the literature. Corresponds to Figs. 4 and 5 in the main text. This table presents the critical moisture values extracted from the literature that were used to generate Figs. 4 and 5, and includes information on temperature, environment, document type, and scenario [95, 91, 96–98]. (DOCX 34 kb) [file 40168_2015_137_MOESM3_ESM.docx]

Table S3: Additional Information for Critical Moisture Values to Prevent Microbial Growth Defined in the Literature. Corresponds to Figures 4 and 5 in the main text.

| **Source*** | **RH (%)** | **ERH** (%)** | **MC (%)** | **Environmental Temperature**  **( °C)** | **Environment** | **Document Type** | **Note** |
| --- | --- | --- | --- | --- | --- | --- | --- |
| [9] | 80 |  |  |  | Field | Guideline | Stated in the introduction as the commonly accepted value that prevents mold growth in buildings. |
| [44] | 97 |  |  |  | Laboratory | Research |  |
| [21] | 65 |  |  |  | Field | Standard | Recommendation for ventilation systems with dehumidification capabilities, not specifically related to microbial growth. |
| [95] |  | 80 |  | 5-40 | Field | Standard | Surface moisture threshold to minimize the risk of microbial growth on indoor surfaces when 30-day running average surface temperature is between 5-40 °C. |
|  |  | 98 |  |  |  |  |  |
|  |  | 99.9 |  |  |  |  |  |
| [87]^1^ | 97 |  |  |  | Laboratory | Research |  |
| [96] | 73 |  |  |  | Field | Guideline | Moisture for mold growth on hygroscopic materials (non-hygroscopic materials require 100% RH). |
| [4] |  |  | 1.4 |  | Laboratory | Research |  |
| [8] |  |  | 1 |  | Laboratory | Research |  |
| [88]^2^ |  |  | 0.6 |  |  | Research | MC for growth on gypsum board without the paper covering. |
| [94] | 60 |  |  |  | Field | Guideline | General recommendation for buildings in hot, humid locations (i.e., tropics, subtropics). |
|  | 80 |  |  |  | Field | Guideline | General recommendation for buildings in cold climates (NA) but states that there is no consensus. |
| [76] |  | 80 |  |  | Field | Research | Defined as a_w_ in the source, but categorized as ERH in this table since ERH and a_w_ are equivalent under equilibrium conditions. |
|  |  | 97 |  | 5 | Laboratory |  |  |
|  |  | 98 |  | 12 |  |  |  |
|  |  | 94 |  | 18 |  |  |  |
|  |  | 93 |  | 25 |  |  |  |
|  |  | 97 |  | 5 |  |  |  |
|  |  | 89 |  | 12 |  |  |  |
|  |  | 90 |  | 18 |  |  |  |
|  |  | 90 |  | 25 |  |  |  |
|  |  | 89 |  | 5 |  |  |  |
|  |  | 89 |  | 12 |  |  |  |
|  |  | 88 |  | 18 |  |  |  |
|  |  | 89 |  | 25 |  |  |  |
|  |  | 87 |  | 5 |  |  |  |
|  |  | 87 |  | 12 |  |  |  |
|  |  | 88 |  | 18 |  |  |  |
|  |  | 86 |  | 25 |  |  |  |
|  |  | 93 |  | 20 |  |  |  |
|  |  | 90 |  | 20 |  |  |  |
|  |  | 90 |  | 20 |  |  |  |
| [20] | 80 |  |  |  | Field | Guideline | Monthly average indoor RH near a surface (i.e., not only gypsum drywall) that should not be exceeded. |
| [91] | 80 |  |  |  | Field | Guideline | Recommendation for buildings, not specifically related to microbial growth or gypsum drywall. |
| [25] | 89 |  |  | 22 | Laboratory | Research | Critical range of 89-95%. 89% is taken as the threshold value. |
|  | 95 |  |  | 10 |  |  |  |
|  | 89 |  |  | 22 |  |  | Critical range of 89-95%. 89% is taken as the threshold value. |
|  | 95 |  |  | 10 |  |  |  |
| [89] | 99 |  |  | 5 | Laboratory | Research | Value extrapolated from growth limit curves: 99-100% at 5 °C. |
|  | 97.5 |  |  | 10 |  |  | Value extrapolated from growth limit curves: 97.5-99% at 10 °C. |
|  | 96 |  |  | 15 |  |  | Value extrapolated from growth limit curves: 96-98% at 15 °C. |
|  | 95 |  |  | 20 |  |  | Value extrapolated from growth limit curves: 95-98% at 20 °C. |
|  | 95 |  |  | 25 | Field |  | Value extrapolated from growth limit curves: 95-98% at 25 °C. |
|  | 95 |  |  | 30 |  |  | Value extrapolated from growth limit curves: 95-98% at 30 °C. |
|  | 95 |  |  | 35 |  |  | Value extrapolated from growth limit curves: 95-98% at 35 °C. |
|  | 96 |  |  | 40 |  |  | Value extrapolated from growth limit curves: 96-98.5% at 40 °C. |
|  | 98 |  |  | 45 |  |  | Value extrapolated from growth limit curves: 98-99.5% at 45 °C. |
| [97] |  | 70 |  | 4.4-37.7 | Field | Guideline | RH limit for mold growth, not specific to gypsum drywall. |
|  | 25 |  |  | 21.1 |  |  | Indoor RH limit to control surface conditions (i.e., not specific to gypsum drywall) for cold climates in the heating season. |
|  | 30 |  |  | 21.1 |  |  | Indoor RH limit to control surface conditions (i.e., not specific to gypsum drywall) for cold and mixed climates in the heating season. |
|  | 40 |  |  | 21.1 |  |  | Indoor RH limit to control surface conditions (i.e., not specific to gypsum drywall) for mixed climates in the heating season. |
|  | 60 |  |  | 23.8 |  |  | Indoor RH limit to control surface conditions (i.e., not specific to gypsum drywall) for hot humid climates in the cooling season. |
| [14] | 97 |  |  | 21.1 | Laboratory | Research | <97% RH, wetting is required. |
| [47] | 90 |  |  |  | Field | Guideline | Stated, but not based on the author's experiments. |
| [86] |  | 70 |  |  | Field | Guideline | Recommended limit for indoor surfaces (i.e., not specific to gypsum drywall). |
| [90] | 70 |  |  | 25 | Laboratory | Research | Ergosterol (which indicates microbial activity) was found on only 1 gypsum drywall sample at 70% RH. Ergosterol was detected on many other samples at 80% and 90% RH. |
| [24] | 86 |  |  | 25 |  |  | Slight colonization on one gypsum sample. No growth detected on gypsum samples at lower RHs at this temperature. |
|  | 86 |  |  | 20 |  |  | Superficial growth only. No growth detected on gypsum samples at lower RHs at this temperature. |
|  | 90 |  |  | 10 |  |  | Growth on wallpapered gypsum drywall (no growth on plain gypsum drywall samples). No growth detected on gypsum samples at lower RHs at this temperature. |
|  | 95 |  |  | 10 |  |  | Heavy colonization. No growth detected on gypsum samples at lower RHs at this temperature. |
|  | 91 |  |  | 5 |  |  | Slight growth. No growth detected on gypsum samples at lower RHs at this temperature. |
| [2] |  | 80 |  |  | Field - controlled | Research | Surface moisture threshold for mold growth in buildings (i.e., not specific to gypsum drywall) without thermal bridges. Determined for real buildings using an algorithm. |
|  | 70 |  |  |  |  |  | Ambient moisture threshold for mold growth in buildings without thermal bridges (i.e., not specific to gypsum drywall). Determined for real buildings using an algorithm. |
|  | 60 |  |  |  |  |  | Ambient moisture threshold for mold growth in buildings with thermal bridges (i.e., most; not specific to gypsum drywall). Determined for real buildings using an algorithm. |
|  | 70 |  |  |  | Field | Guideline | Defined as the generally accepted moisture limit for buildings. |
| [64] | 82 |  |  |  | Laboratory | Research |  |
| [56] |  |  | 30 | 22-25 | Laboratory | Research | MC for dense colonization. |
|  |  |  | 25 |  |  |  | MC for sparse growth. |
|  | 95 |  |  |  |  |  | Growth on inoculated gypsum drywall samples. |
|  | 95 |  |  |  |  |  | Growth on inoculated gypsum drywall samples after sanitation, cleaning, and application of a coating. |
| [92 | 97 |  |  | 15 | Laboratory | Research | RH in control chamber. |
|  | 90 |  |  | 23 |  |  |  |
| [52] | 80 | 80 |  |  | Field | Guideline | Growth can occur when there is a local RH of 80% (not specific to gypsum drywall). |
| [59] |  | 79 |  |  | Laboratory | Research | ERH whereby no growth was observed after 1440 hours for gypsum samples with Czepak additive that achieved a desired ERH via soaking in an aqueous solution. |
| [98] |  | 65 |  |  | Field | Guideline | Localized humidity at interior surfaces to prevent microbial growth. Not specific to gypsum drywall. |

*Where a range of critical moisture values was stated in the literature, the lower value was taken as the critical value for this analysis (since the onset of growth could occur at the lowest value).

**Measurements of a_w_ have been categorized under ERH because the two are equivalent under equilibrium conditions.

^1^ As cited in [9].

^2^ As cited in [94].
